# Colorectal Cancer and Asbestos Exposure: A Women’s Health Perspective

**DOI:** 10.3390/healthcare12181816

**Published:** 2024-09-11

**Authors:** Antonietta Porzio, Alessandro Feola, Cecilia Salzillo, Graziamaria Corbi, Carlo Pietro Campobasso

**Affiliations:** 1 Department of Mental and Physical Health and Preventive Medicine, University of Campania “Luigi Vanvitelli”, Via Luciano Armanni 5, 80138 Naples, Italy; antonietta.porzio@unicampania.it; 2 Department of Occupational and Environmental Medicine, Epidemiology and Hygiene, National Institute for Workers’ Compensation (INAIL), 00143, Rome, Italy; 3 Department of Experimental Medicine, University of Campania “Luigi Vanvitelli”, Via Luciano Armanni 5, 80138 Naples, Italy; carlopietro.campobasso@unicampania.it; 4 Department of Precision and Regenerative Medicine and Ionian Area, Pathology Unit, University of Bari “Aldo Moro”, Piazza Giulio Cesare 11, 70121 Bari, Italy; cecilia.salzillo@unicampania.it; 5 Department of Translational Medical Sciences, University of Naples “Federico II”, 80138 Naples, Italy; graziamaria.corbi@unina.it

**Keywords:** asbestos, colon cancer, asbestos exposure, occupational cancer, female

## Abstract

Background: Colorectal cancer (CRC) is considered a “man’s disease”. However, emerging data show that females may have a higher prevalence of certain risk factors. A potential causal role of asbestos in CRC carcinogenesis has been suggested. This relationship is controversial, and only a few studies have focused on exposed female populations. The aim of this study was to review the scientific literature related to asbestos-related CRC incidence and mortality rates in female populations to address gender bias in the existing research. Methods: A systematic review was performed following PRISMA statement. Results: Fourteen studies reporting 92 cases in total were included. Most women were aged 50 years or older and were employed in occupational activities with high asbestos exposure (steel, textile, and asbestos-cement industry) for at least 10 years. In one single case, household asbestos exposure was reported. The colon was the primary location of the tumor in 47 out of 92 cases. Three women were also affected by synchronous or metachronous peritoneal mesotheliomas. Conclusions: This study revealed a general methodological “gender bias” in scientific research. A significantly higher representation of women in clinical studies is needed to clarify the link between asbestos exposure and the development of colorectal cancer.

## 1. Introduction

Colorectal cancer (CRC) is the second leading cause of cancer related deaths in both genders worldwide [1] with 515,637 deaths among males and 419,536 deaths among females in 2020. The global CRC incidence rate in women is 15.2 cases per 100,000 persons, and the age-standardized mortality rate is per 6.5 per 100,000 person-year; the corresponding rates for the male population are higher (21.9 per 100,000 person-year and 9.9 per 100,000 person-year) [2]. In Italy, in 2020, approximately 43,700 new CRC diagnoses were reported, among which 20,300 cases occurred in women resulting in 10,200 deaths in total. There are 280,277 men and 233,200 women currently living in Italy after a diagnosis of CRC [3].

Several researchers have identified male gender as a potential risk factor to the onset of CRC [4,5]. However, other studies have shown minor differences between males and females in terms of screening acceptance, pathway to diagnosis, cancer stage at diagnosis, and survival rates [6]. Although the risk of CRC is quite comparable between men and women, there is a common perception among women that CRC is primarily a “man’s disease” [7]. The perception of a low risk of CRC may influence lower participation in CRC screening among women [8]. Embarrassment and fear of discomfort for endoscopic screening could represent other reasons for the reduced adherence. Furthermore, the symptoms of CRC can be mistaken for common pelvic sensations especially for individuals with pathological or physiological conditions like endometriosis or perimenopause, menopause, and postmenopause. Fatigue, pelvic pain, lower back pain, cramping, bloating, and migraine could mimic CRC symptoms. This may lead to a delay in diagnosis and allow the cancer to progress to an advanced stage. CRC mortality rates are higher in women aged over 65 compared to men in the same age group [9], and the 5-year survival rate is lower for women over 70 years old [10].

CRC risk factors include advanced age, inflammatory bowel diseases (such as Crohn’s disease), family history, Lynch syndrome, physical inactivity, and specific dietary habits. Notably, females may have a higher prevalence of certain risk factors, such as overweight or obesity [11], particular inflammatory bowel diseases like Crohn’s [12] and specific genetic mutations [13] associated with colon cancer. A recent study suggests an increased risk of CRC among individuals with gynecological cancers, particularly those with endometrial and ovarian cancers. Patients with Hereditary Nonpolyposis Colorectal Cancer (HNPCC) have an increased chance of developing endometrial (40–60%) and ovarian cancer (1.5–10%) [14,15]. Furthermore, individuals diagnosed with gynecological cancer may benefit from colonoscopy screening [16].

The etiopathogenesis of CRC is not clear, but a potential carcinogenetic role of asbestos was suggested [17,18]. While there is wide agreement on the carcinogenic effects of asbestos leading to lung cancer and mesothelioma, conflicting opinions exist regarding other cancer sites. According to the 2012 International Agency for Research on Cancer (I.A.R.C) Monographs on asbestos, larynx and ovarian cancer are also assessed as associated with sufficient evidence in humans (Group 1) with asbestos exposure [19]. A recent meta-analysis [20] contributed to confirm that the risk of ovarian cancer roughly doubles in women with occupational exposure to asbestos. For CRC, stomach, and pharynx tumors, only a “positive association” with asbestos has been established, and the relationship between asbestos exposure and CRC remains a controversial topic. Although some studies suggest an exposure–response relationship between CRC and fiber exposure, no increased risks of developing bowel cancer in asbestos-exposed workers have been documented by others [21,22]. Focusing on CRC by anatomical location in asbestos-cement workers, an increased incidence of malignancy in the right part of the colon, but not in the left side, has been observed by Jakobsson et al. (1994) [23]. This is worth mentioning as right-sided colon cancer is more prevalent in women than in men, and it is associated with a more aggressive form of neoplasia compared to left-sided colon cancer. Several other studies have also documented that CRC could be associated with synchronous or metachronous peritoneal mesothelioma both in males and females with a documented asbestos exposure [24].

The scientific community has predominantly examined incidence and mortality rates of CRC in asbestos-exposed male populations. The overall goal of this study, then, was to review scientific literature on CRC incidence and mortality rates linked to asbestos exposure in female populations while addressing the notable gender bias present in the existing research. Further research is still needed to clarify the role of asbestos in CRC pathogenesis in both genders. 

## 2. Materials and Methods

A systematic review of the scientific literature was independently conducted by two examiners according to the PRISMA (Preferred Reporting Items for Systematic Reviews and Meta-Analyses) statement’s criteria for new systematic reviews, which included searches of databases, registers, and other sources [25]. 

### 2.1. Selection of Studies 

A keyword search for “colorectal cancer AND asbestos” was performed in all fields of the electronic databases PubMed, Scopus, and Web of Science to identify pertinent research available up to 10 December 2023, without time limits. Two authors screened the articles based on the title and abstract and read the full text to evaluate their eligibility according to the inclusion and exclusion criteria. The title, abstract, and full text of each study were reviewed. In addition, the reference lists of the included studies were reviewed manually. Disagreements on the eligibility of studies were solved through a preliminary discussion between the two examiners. If no agreement could be reached, it was resolved using a third co-author as reviewer.

### 2.2. Inclusion and Exclusion Criteria

The inclusion criterion was epidemiological studies or case studies of women exposed to asbestos with a diagnosis of CRC. The exclusion criteria were the following: (1) manuscripts not published in English, (2) studies with unavailable full texts, (3) studies exclusively focused on males, (4) studies that unified both gender groups in the analysis of the results, and (5) studies on animal models. 

### 2.3. Data Extraction

For each eligible study, the following data were collected: the first author, publication year, country, sample size, number of females, age, type of asbestos exposure (occupational, familial, environmental), asbestos exposure time (the time-period from the beginning of the occupational exposure to the end of the work at risk) in cases of occupational exposure, histopathological characteristics of CRC (staging, grades), and other risk factors for CRC. 

## 3. Results

The search of PubMed, Scopus, and Web of Science databases provided 411 articles in total: 122 articles from PubMed, 151 articles from Scopus, and 138 articles from Web of Science. After adjusting for duplicates, 143 studies were discarded. After reviewing titles and abstracts, 229 studies were discarded since they did not satisfy the inclusion criteria. A total of 39 reports were identified as potentially relevant and were sought for retrieval; out of these reports, 3 could not be retrieved. After the addition of 20 articles successfully retrieved and assessed for eligibility from the references, a full-text assessment of 56 articles was performed. One manuscript was not in English, and a second one was on animal models. In nine manuscripts, there was not enough information. Five articles were classified as reviews or metanalyses. For 10 manuscripts, no gender differences were applied, and in 16 manuscripts, the study was restricted to male subjects only. 

**Figure 1:**
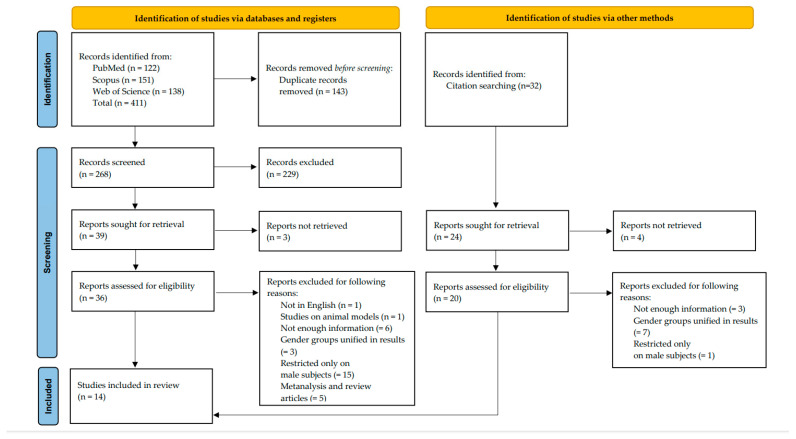
Flowchart depicting the selection of studies for the systematic review.

Finally, only 14 studies satisfied the inclusion criteria, reporting in total 92 cases of asbestos-exposed females suffering intestinal cancer. Figure 1 shows a flowchart depicting the selection of studies according to PRISMA standards. 

Details of the 14 studies are summarized in Table 1. They are mostly represented by cohort studies (10 out of 14). For each type of study, the sample size, age groups, anatomical location of CRC, occupational history, duration of exposure, and risk factors (where available) were reported. All the ten cohort studies have explored the potential risks of CRC linked to occupational exposure to asbestos in female populations. Additionally, two case reports and two case series were also examined in the review. The studies were mostly conducted in Europe (Italy, Austria, France, Germany, Great Britain, Poland, Finland) between 1985 and 2023. A case report was published by a group of Saudi Arabian researchers in 2023. Nine studies involved both males and females, and five involved females only. A total of 5792 female workers have been reported by 11 articles. 

Four studies were conducted in an asbestos textile industry and two in asbestos cement plants. Other studies included cohorts of workers in mining, the steel industry, the manufacture of fireproof textiles, and friction materials plants. One study [29] was not targeted at specific industries but involved cohorts of asbestos-exposed workers in several industries. A cohort study [27] involved the wives of asbestos cement workers with domestic asbestos exposure. Unfortunately, in a case report [38], the occupational exposure was not available. In a study investigating the previous possible exposure to asbestos fibers [38], it was found that the patient had for many years an asbestos shield around his living room heater. 

Four studies [30,31,33,38] did not provide information about the duration of exposure. In the other studies, the time of occupational exposure ranged from the minimum of 2 years to the maximum of 38 years. The mortality rate of workers employed for at least three months at an asbestos plant producing yarn, cloth, cords, packings, brake linings, and asbestos-rubber sheets has been investigated in Finland via a cohort study [28]. Several studies have analyzed the exposure levels to asbestos. Pira et al. [36] reported an Italian cohort of asbestos textile workers with a heavy asbestos exposure to various types of fibers (mostly crocidolite) at levels higher than 100 fb/mL, based on measurements taken from carding departments. Boulanger et al. [35] divided the sample study into two exposure groups: two women with a CRC were included in the lowest exposure group (with a cumulative exposure index ≤ 80 fibers/mL × years), and four cases of CRC were observed in the group with a higher cumulative exposure (> 80 fibers/mL × years). 

The studies included in this review report 92 cases of CRC in total. The main anatomical location of the tumor was the colon (47 out of 92 cases). In 11 out of 92 cases [31], the specific site of the tumor was not provided and was classified as “intestine and rectum” according to codes 152 (Malignant neoplasm of small intestine, including duodenum), 153 (Malignant neoplasm of colon), and 154 (Malignant neoplasm of rectum, rectosigmoid junction, and anus) of the Ninth Revision of the International Classification of Diseases (ICD-9). Clin et al. (1999) used the codes provided by the 3rd edition of international classification of diseases for oncology (ICD-O 3) to describe each anatomical site of cancer [34]: 14 cases of “Colon Rectum” neoplasms were reported. Rectal cancer was assessed in fifteen subjects, and only one case showed a sigmoid colon cancer [38]. In seven studies, the subject’s age was not reported. For the remaining articles, the youngest subject was 55 years old, while the oldest one was 83 years old. The mean age of the overall cohorts reported by Clin et al. (1999) and Boulanger et al. (2015) was 38.7 (SD 12.5) years and 39.1 (SD 13.29), respectively [34,35]. Only four studies [26,29,37,38] provided information about potential risk factors (including a family and personal medical history of cancer and smoking).

### 3.1. The Cohort Studies

The largest cohort study is provided by Peto et al. (1985) [26]. The authors focused on the mortality rate of 3639 workers employed at a Rochdale asbestos textile plant in comparison with the expected national and local rates at both the national and Rochdale levels. Three distinct groups of workers (145 men employed before 1933, 283 women employed in 1933 or later, and 3211 men employed between 1933 and 1974) were monitored until 30 June 1983. The information needed for following up the women of the second group were ascertained by matching the original factory’s records against the National Health Service Register held by the Office of Population Censuses and Surveys at Southport. Because of the small number of observed deaths, this study has limited value for describing or assessing cancer risk. However, it is noteworthy that the authors reported an excess mortality due to CRC in women who were first employed before 1950 [3 against the 1.58 expected; the standardized mortality ratio (SMR) was 1.90]. A single death from CRC was reported in a woman who was employed after 1950.

In 1993, Magnani et al. [27] focused on cancer mortality rates among the wives of asbestos cement workers in Casale Monferrato, northwest Italy. In 1964, women were included in the cohort study: 1740 had domestic asbestos exposure, and 224 married an asbestos worker after they had stopped the asbestos-exposed work activity. Analyses were limited to women under 79 years old to reduce death cause misclassification. From 1965 to 1988, 210 deaths occurred among exposed women: there were 14 CRC deaths, compared to an expected number of 9.9 (SMR 141.2; 95% CI 77.2–236.9).

Meurman et al. [28] provided information about 736 male and 167 female workers at two anthophyllite mines in Finland. The authors divided the cohort study into two groups: “heavily exposed” workers, who had been employed in the mines or the mill, and “moderately exposed” workers, who comprised the rest of the personnel. In the subset of 98 women with significant exposure, there were 16 cancer cases against the 10.7 expected. Most of this excess was attributed to endometrial cancer, primarily observed in the oldest age and longest follow-up period since the first employment groups of women. However, among the heavily exposed female workers, two cases of CRC and one case of rectal cancers were also identified.

A cohort study involving 616 female German workers with a history of asbestos exposure was conducted by Rösler et al. between 1977 and 1988 [29]. During the observation period, 64 women passed away. The average age at which the participants were first exposed to asbestos was 32 years, ranging from 14 to 59 years, and the average length of exposure was 12 years. To be included in the study, participants were required to have a minimum of 3 years and a maximum of 43 years of employment. The mean interval period since the onset of exposure was 29 years, ranging from 11 to 65 years, and the mean interval period since the cessation of exposure was 15 years, ranging from 1 to 54 years. The results also showed that the female mortality rates for lung cancer and mesothelioma were twice and four times higher, respectively, than those observed in men. Three deaths due to CRC were also reported, which is less than the 3.1 deaths expected, with an SMR of 0.96 (95% CI 0.20–2.81). Therefore, the authors concluded that women exposed to asbestos in Germany belong to a high-risk group for developing mesothelioma and lung cancer, underlining the need to improve cancer screening, secondary prevention, and rehabilitation in exposed women. 

The 1999 cohort study published by Germani et al. [31] comprised 631 Italian women who were compensated for asbestosis and had significant occupational exposure to asbestos. The National Institute for Insurance of Occupational Accidents data were used to define the sample study. For the deceased subjects, the causes of death were obtained from the Registry Office of the municipalities of residence or of death. The mortality rate for all causes, as well as CRC, was significantly higher among women compensated for asbestosis. Exposed workers reported an increased mortality for malignant neoplasms of the digestive system with an SMR of 173 (95% CI 118–245). Following the Ninth Revision of International Classification of Diseases (ICD-9) the authors reported 8 cases of colon and sigma tumors (SMR = 238; 95% CI 103–470), 11 cases of intestine and rectum cancers (SMR = 218; 95% CI 109–390), and one rectum cancer (SMR = 62; 95% CI 2–345). Two separate analyses were conducted, each focusing on a specific industry: textiles and asbestos-cement. According to the results, women in the textile industry, mainly exposed to chrysotile, were compensated for asbestosis at a younger age and showed a higher SMR for digestive colon and sigma cancers. The research also provides insights on the relationship between asbestos exposure and an increased risk of CRC in occupationally exposed workers, among whom the authors considered the possible misclassification of peritoneal mesothelioma as a contributing factor to CRC.

Berry et al. [32] focused on the mortality rate of individuals who had worked at an asbestos factory in the East End of London. A total of 700 women hired between 1936 and 1942 were followed up until June 1980. The sample study was divided into two exposure categories (low-to-moderate versus severe). Severe exposure to asbestos occurred in workers involved in making sectional pipes for the production of insulating material with a high asbestos content, those in the textile and mattress sections, openers, disintegrators, and individuals responsible for the disposal of dust. A substantial excess and a statistically significant exposure–response relationship has been reported for the colon site (ICD-7 153; RR 1.8, 95% CI 1.2 2.7, *p* = 0.017). However, the excess risk of colon cancer was limited to male workers who had served as laggers or had experienced prolonged, severe exposure for over two years.

The Polish study of Wilczyńska et al. [33] examined the mortality rate among workers at an asbestos plant producing yarn, cloth, cords, packings, brake linings, and asbestos-rubber sheets. The sample study included 4497 workers (3027 males and 1470 females) employed for at least three months from 1945 to 1980. Overall, 60.7% of male workers showed shorter employment durations than female workers (41.5%). Most workers (72.6%) were under 40 years when they began working. The SMR for female workers was 136 (95% CI 124–149), indicating a significantly higher mortality compared to the reference population. Among the 435 deaths occurred in female workers, 135 were due to malignant neoplasms, primarily gallbladder cancer, lung cancer, pleural mesothelioma, and cervical cancer. In the insulation material departments, there were three cases each of colon and rectal cancer, while the yarn, cloth, or auxiliary departments had two cases of colon cancer and four of rectal cancer.

In 2009, Clin B. et al. [34] published the results of a retrospective morbidity study consisting of 2024 individuals who had previously worked at an asbestos reprocessing plant located in Normandy, Southern Calvados, for at least one year. A total of 420 women with an average age of 39.1 years and a mean duration of employment of 16.6 years [15.4–17.7] were included. In the population study, 85 cases of digestive cancer were observed in total from 1978 to 2004. Overall, 12.94% of these victims were female, and 4 cases of CRC were observed versus the 3.86 expected. In both genders, cumulative exposure indexes for asbestos inferior or equal to 80 fibers/mL × years were reported, and no significant elevated incidence of digestive cancer was observed.

Boulanger et al. (2015) [35] analyzed the incidence of digestive cancers within a cohort of 2024 asbestos-exposed workers of an asbestos reprocessing plant. A total of 419 females in total were included in this study. The mean duration of the follow-up was 22.3 years, with a standard deviation of 8.93 years. A mean duration of work at inclusion of 15.9 years (S.D. 9.1) was also reported for 334 participants already employed in the plant in 1978. The exposure assessment classified workers having a cumulative exposure index (CEI) ≤ or ≥ 80 fibers/mL × years, an exposure duration ≤ or ≥ 25 years, and a mean level of asbestos exposure ≤ or ≥ 4 fibers/mL. The standardized incidence ratio (SIR) for CRC cancers for women was 1.33 (95% CI 0.49–2.91) based on the female population of Calvados (France) as the reference for expected cancers. Four out of six cases of CRC were identified in women who belonged to the highest exposure class, characterized by a CEI of ≥ 80 fibers/mL × years. However, five CRC cases were also observed in the lower exposure duration class. The authors found a relationship between asbestos exposure and CRC only for the male population, while for women, an increased risk was observed for peritoneal mesothelioma only.

Pira et al. (2016) followed up a cohort of 1083 female and 894 male textile workers employed in an asbestos textile factory in Northern Italy between 1946 and 1984, with heavy asbestos exposure up to 100 fb/mL [36]. A total of 1019 deaths were reported, resulting in an increased SMR for lung, ovarian, peritoneal, and pleural cancers. The data showed that there were 9 cases of CRC among females and 24 cases among males, with a non-statistically significantly increased SMR of 0.93 (95% CI 0.43–1.77) and 1.73 (95% CI 1.11–2.58) respectively. According to the authors, the lack of statistical significance in this study may be due to the rarity of these cancers in women, making it difficult to draw causal conclusions regarding CRC.

### 3.2. The Case Reports and Case Series Studies

The four remaining studies are discussed only briefly. Jatzko et al. [30] published a case of a 55-year-old woman with rectal bleeding. A diagnosis of rectal carcinoma was later assessed, when peritoneal seedings in the major and minor omentum, visceral and parietal peritoneum, and both diaphragm halves had already occurred. The patient was diagnosed with a simultaneous diffuse well-differentiated papillary mesothelioma of the peritoneum. After the surgery, it was discovered that the patient had been exposed to asbestos for a prolonged period due to the presence of an asbestos shield around a living room heater at home. Serio et al. reported an unusual case of a 58-year-old woman with an history of occupational asbestos exposure who developed a rectal adenocarcinoma and, after four years, a metachronous epithelioid mesothelioma [37]. No asbestos fibers were detected in rectal adenocarcinoma samples. Since 1971, she had worked as a dressmaker in a textile company. After suspending the activity for 5 years, in 1980 she started working for the same employer again for another decade. According to the family medical history, her father’s laryngeal cancer was reported. No CRC risk factors were found. A case of synchronous malignant peritoneal mesothelioma and sigmoid adenocarcinoma in a 75-year-old woman was presented by Alratrout et al. in 2023 [38]. No clear history of asbestos exposure was reported by the patient. She only reported a significant family history of cancer: her father passed away after being diagnosed with a brain tumor, and her daughter had been diagnosed with thyroid cancer. Finally, Porzio et al. [39] presented a case series of 35 cases of colorectal cancer in asbestos-exposed workers, which included two female maintenance workers aged 77 and 83 years, respectively. These workers had been exposed to asbestos for 27 and 22 years while working at a steel company. Unfortunately, the limitations of this retrospective study were the small sample size and the inability to determine the cumulative asbestos exposure.

## 4. Discussion

The relationship between exposure to asbestos and CRC have been investigated by several reviews and metanalyses [40,41,42]. However, the etiology of CRC is still unclear, and the underlying factors have yet to be fully explained. 

The 2012 I.A.R.C Monographs on asbestos reported a positive link between asbestos exposure and the development of CRC based on 41 occupational cohorts and 13 case–control studies [19]. However, the available evidence was not considered strong enough by the IARC Working Group to categorize this relationship as Group 1 (with sufficient evidence in humans). An association between CRC and asbestos was found to be limited in human studies and therefore categorized as Group 2A (with limited evidence in humans). Unfortunately, the sample study in most of the research studies reviewed by the IARC Monographs Volume 100C was largely represented by males alone. Cases of CRC in females exposed to asbestos are poorly reported. 

The results of our systematic review show that only 14 out of 435 studies focused on women exposed to asbestos with a diagnosis of CRC. Most studies have focused on male participants or failed to investigate gender disparities; in certain studies, women were initially included in the sample, but were subsequently excluded from the results. The potential risk of CRC among female workers exposed to asbestos has been examined in only 10 analytical epidemiologic studies since 1985. Four studies documented a total of five CRC cases in women who had been exposed to asbestos. The asbestos exposure was assessed for 13 out of the 14 studies: 4 studies were conducted in the asbestos textile industry [26,29,31] and 2 in asbestos cement plants [32,33], and others involved cohorts from mining, the steel industry, and the manufacture of fireproof textiles and friction materials. Occupational exposure to asbestos ranged from a minimum of 2 to a maximum of 38 years. In one case, a patient revealed long-term exposure to an asbestos shield around his living room heater [30]. Notably, three cases of CRC arose in asbestos-exposed women, two of whom were affected by synchronous mesothelioma [30,38] and one of whom developed metachronous mesotheliomas [37]. This co-occurrence of peritoneal mesothelioma and CRC is interesting and could represent a topic worthy of further investigation in future studies. Unfortunately, among the cohort studies, only two studies reported an increased SMR among the exposed female workers [26,31]. However, the small sample size represents a significant limitation affecting the generalizability and validity of these study’s findings. 

Gender bias permeates all areas of scientific research, mainly in relation to the selection of the study samples often being represented by males, as also observed in the manuscripts analyzed in this systematic review. This is the main confounding factor in gender medicine and research and also affects data analysis and interpretation [43]. The “androcentric approach”, treating males as the norm for human beings, has led to a severe underrepresentation of women and minorities in science. Gender medicine has received considerable attention in recent years due to the substantial and relevant disparities observed between men and women in health and illness. However, gender inequality in occupational safety and health is still concerning [44]. Against longstanding gender disparities in employment, rooted in social and historical factors, women’s workforce participation is rising. The guidelines of the European Agency for Safety and Health at Work (EU-OSHA) also recommend incorporating gender considerations into occupational safety and health (OSH) practices [45,46,47,48] by considering biological (sex) and socio-economic (gender) differences among workers. The research on women in occupational settings has concentrated on gender inequalities, work organization hazards, and psychosocial stressors, with fewer toxicological and physiological studies. Very few studies have examined sex susceptibility to hazardous substances and biological agents [44]. Chemical and biological work-related risks in female populations are poorly investigated. 

Historically, asbestos-related diseases have primarily affected exposed male workers. Therefore, asbestos-related health issues have not been as strongly linked to women. Nevertheless, mesothelioma and other asbestos-related diseases cannot be considered just a “men’s disease”. Already in the 1960s, two English studies on asbestos-exposed populations concluded that there was a significant occurrence of peritoneal tumors in female patients suffering with asbestosis [49,50]. A total of 900 female workers hired in an asbestos factory from 1936 to 1942 were involved in a cohort study revealing an excess of lung, pleural, and other cancer deaths for individuals with severe exposure for more than two years [51]. According to other studies, asbestos can accumulate in the ovaries of women exposed to asbestos both in their households [52] and during occupational activities [53]. A possible association between perineal talc use and the development of ovarian cancer has been reported in several studies that have reported the presence of asbestos fibers in cosmetic talc [54,55].

According to the Italian epidemiological surveillance system ReNaM (Italian National Registry of Malignant Mesothelioma), in the period between 1993 and 2018, 31,572 cases with a diagnosis of malignant mesothelioma (MM) were collected. The exposure evaluation was assessed for 24,864 cases: among them, 2232 women had an occupational exposure to asbestos [56]. Marinaccio et al. analyzed the main occupational exposure for female mesothelioma incident cases [57]: the textile industry and manufacturing of wearing apparel, asbestos-cement, and asbestos-textile industries, as well as other industrial settings, such as the chemical, plastic, and food and beverage industries. Hairdressers, dressmakers, farmers, clerks, and teachers could be exposed to asbestos fibers. According to the latest ReNaM report (2021), women comprised the majority of non-occupationally exposed MM cases. Specifically, a proportion of 29% of women who experience MM due to non-occupational exposure (such as exposure from family or the environment) has been reported versus 4.4% of the corresponding male sample [58]. Soiled work clothes brought home by occupationally exposed relatives could play a significant role in fibers transmission. It is important to consider this pathway when assessing the risk of familial exposure for female MM patients [59]. The 2022 report by the Italian National Institute of Health shows a total of 4410 deaths per year, from 2010 to 2016, due to asbestos exposure, among which 550 victims per year were females [54]. In this five-year retrospective analysis, 2947 females died due to mesothelioma, with a national mortality rate of 1.11 per 100,000 women, and 44 women exposed to asbestos died from asbestosis, 672 from lung cancer, and 96 from ovarian cancer. These latter victims were part of the total 22,465 females who died due to ovarian neoplasms. 

The potential risk of the underestimation of asbestos-related deaths in female populations must be considered. The wide range of jobs and domestic situations that could potentially involve asbestos exposure, along with the challenges in identifying occupational exposure, highlight the need for implementing tools such as the anamnestic questionnaire to investigate exposure modalities from a gender perspective. This literature review has revealed a significant gap in gender medicine dealing with asbestos and CRC. These disparities are also evident in other areas such as education, employment, health, and social status. Interventions to address gender inequities and promote equality are needed. Improving the awareness among clinicians and healthcare operators of the occupational or environmental origin of asbestos-related diseases in women could enhance the effectiveness of the insurance and welfare system, and support prevention policies for exposure risks.

## 5. Conclusions

The results of this systematic review do not provide evidence enough to determine a relationship between CRC and asbestos exposure in females. Only two cohort studies reported an increased SMR for CRC among the exposed female workers. Other studies suggest that women have an equal likelihood of developing an asbestos-related disease from environmental and/or professional exposure. Unfortunately, most of the scientific research is focused on males, and women are significantly under-represented. Very few studies reported sex-specific CRC risk estimates. Gender bias in research due to systematic discrimination can affect influence the quality, validity, interpretation, and applicability of the results. Further research addressing sex-specific differences in responses to asbestos for CRC is required. This can provide more information to understand gender-related biological and socio-cultural differences and to define specific strategies for screening, treatment, and prevention protocols to reduce mortality rates and increase the quality of life.

## Figures and Tables

**Table 1:** Details of the 14 studies (sample size, age groups, anatomical location of CRC, occupational history, duration of exposure, and risk factors).

References	Type of Study	Country	N. Subjects	Female	N. of Cases and Cancer Site (C = Colon R = Rectum S = Sigmoid)	Age	Occupational Exposure (F = Factory)	Years of Exposure	Risk Factors
Peto et al. 1985 [26]	Cohort study	Great Britain	3639	283	4–C	n.d.	asbestos textile F	191 cases: 17 92 cases: 24	Smoking
Magnani et al. 1993 [27]	Cohort study	Italy	1964	1964	14 C and R ^1^	<79	not occupational	>10	n.d.
Meurman et al. 1994 [28]	Cohort study	Finland	903	167	6–C 2–R	n.d.	asbestos miners	>3 months	n.d.
Rösler et al. 1994 [29]	Cohort study	Germany	616	616	3–C	n.d.	various (most asbestos textile)	12	smoking
Jatzko et al. 1997 [30]	Case report	Austria	1	1	1–R	55	not occupational	n.d.	n.d.
Germani et al. 1999 [31]	Cohort study	Italy	631	631	11–intestine/rectum ^2^ 8–C 1–R	n.d.	textile and asbestos-cement F	n.d.	n.d
Berry et al. 2000 [32]	Cohort study	Great Britain	5100	700	3–C 4–R	n.d.	asbestos F	< or > 2 of severe exposure	n.d.
Wilczyńska et al. 2005 [33]	Cohort study	Poland	4497	1470	5–C 7–R	n.d.	asbestos F	n.d.	n.d.
Clin et al. 2009 [34]	Cohort study	France	2024	420	4 C and R ^3^	39.1 ^4^	asbestos reprocessing F	≤25	n.d.
Boulanger et al. 2015 [35]	Cohort study	France	2024	419	6–C	38.7 ^5^	asbestos reprocessing F	~16 ^6^	n.d.
Pira et al. 2016 [36]	Cohort study	Italy	1977	1083	9–C	n.d.	asbestos textile F	38	n.d.
Serio et al. 2022 [37]	Case series	Italy	4	1	1–C	58	dressmaker	15	Familial cancer
Alratrout et al. 2023 [38]	Case report	Saudi Arabia	1	1	1–S	75	n.d.	n.d.	Brain and thyroid malignancy
Porzio et al. 2023 [39]	Case series	Italy	35	2	2–C	1: 77 2: 83	steel F	case #1: 27 case #2: 22	n.d.

^1^ Coded according to the eighth revision of the International Classification of Disease (ICD-8). ^2^ Coded according to the ninth revision of the International Classification of Disease (ICD-9). ^3^ Coded according to the international classification of diseases for oncology—3rd edition (ICD-O 3). ^4^ Mean age (SD 13.29). ^5^ Mean age at inclusion (SD 12.5). ^6^ Mean duration of work at inclusion (SD 9.1).

## Data Availability

No new data were created or analyzed in this study. Data sharing is not applicable to this article.
